# Richter transformation to classical Hodgkin lymphoma following recurrent disseminated histoplasmosis in chronic lymphocytic leukemia: a Case Report

**DOI:** 10.3389/fonc.2026.1714656

**Published:** 2026-02-16

**Authors:** Delour Haj, Mohamed Said, Talal Al-Assil, Steven Stone, Mohammad Omaira

**Affiliations:** 1 Western Michigan University Homer Stryker M.D. School of Medicine, Kalamazoo, MI, United States; 2 Hematology & Medical Oncology, Bronson Cancer Center, Kalamazoo, MI, United States

**Keywords:** case report, chronic lymphocytic leukemia (CLL), histoplasmosis, Hodgkin’s lymphoma, Richter transformation

## Abstract

**Background:**

Richter Transformation (RT) is the rare progression of chronic lymphocytic leukemia/small lymphocytic lymphoma (CLL/SLL) into a more aggressive lymphoma, occurring in only about 5-10% of cases. RT has a poor prognosis with rapid progression and resistance to standard therapies. PET/CT scans are crucial in detecting RT with increased SUVmax indicating more aggressive disease.

**Case presentation:**

A 65-year-old male with 13q deletion CLL developed three relapses over the course of 13 years from initial CLL diagnosis, and work up of the third relapse revealed disseminated histoplasmosis. He improved with Ibrutinib for CLL and Amphotericin B for histoplasmosis. Four and seven years later, the patient re-presented with histoplasmosis reinfections, confirmed by PET/CT and biopsy, and treated with antifungals for each reinfection. One year later (21 years from initial CLL diagnosis), he presented with malignant hypercalcemia and retroperitoneal lymphadenopathy (LAD) for which biopsy revealed RT to classic Hodgkin’s Lymphoma (cHL). Given his impaired renal function, his treatment regimen included AVD chemotherapy and nivolumab, from which he eventually recovered.

**Discussion/conclusion:**

CLL patients are inherently immunocompromised due to hypogammaglobulinemia, increasing their susceptibility to opportunistic infections like histoplasmosis. These infections can recur, be refractory, and may even coincide with RT. This unusual case of RT after chronic disseminated histoplasmosis reinfections involved lymph nodes and eventually hypercalcemia. Vigilant monitoring and patient education on risk avoidance are recommended strategies for mitigating risk of histoplasmosis. Providers may also consider Itraconazole prophylaxis. Further research is warranted to better understand the relationship between chronic opportunistic infections and the oncogenic progressions in CLL patients.

## Introduction

Richter Transformation (RT) refers to the rare evolution in 5-10% of chronic lymphocytic leukemia/small lymphocytic lymphoma (CLL/SLL) cases into a more aggressive form of lymphoma (1–3). RT typically signifies the onset of diffuse large B-cell lymphoma carrying a poor prognosis, rapid disease progression, a high rate of resistance to conventional therapies, and mortality. RT can be clonally related or unrelated to the original CLL clone, with clonally unrelated cases often demonstrating a more favorable prognosis (4).

Adequate monitoring of CLL/SLL disease progression via Positron Emission Tomography/Computed Tomography (PET/CT) is key in detection of a RT. PET/CT also assists in guiding biopsy by identifying the most metabolically active lymph nodes, improving diagnostic yield. While PET/CT is valuable in detecting RT, its use is generally reserved for patients with clinical suspicion of transformation rather than routine surveillance. By measuring the standardized uptake values (SUV) in regions of interest, heightened maximum standardized uptake value (SUVmax) can highlight regions with malignant metabolic activity, and thus allow for differentiation of the more aggressive lymphomas from other diseases like CLL or adverse effects of its chemotherapy (5). In fact studies have shown that CLL patients with RT exhibit a mean SUVmax about four times higher than those without transformation. Additionally, CLL patients with high SUVmax values have inferior survival and increased likelihood of developing a RT (5–7). Notably, elevated SUV values may be seen in patients receiving B-cell receptor inhibitors without evidence of RT, limiting the specificity of PET/CT in this context (8).

Furthermore, the immunodeficiency in CLL affects both adaptive and innate immunity, including B-cells, T-cells, neutrophils, and complement pathways; leading to opportunistic infections, which are prevalent across hematologic malignancies (7). Manifestations of the latter can vary, including acute or chronic pulmonary issues, mediastinal fibrosis or granulomas, or disseminated histoplasmosis (9). Most histoplasmosis cases are mild, and sometimes they are asymptomatic, with individuals’ innate immune systems effectively being able to eradicate infection (10). However, chronic or disseminated histoplasmosis can target the reticulohistiocytic system in immunocompromised individuals- causing multi-organ damage (11). Symptoms may be systemic such as fever, weight loss, anorexia, cough, vomiting, diarrhea, and abdominal pain. The recommended methods for diagnosing disseminated histoplasmosis are detection of Histoplasma antigen in blood or urine, culture of H. capsulatum from blood, bone marrow, or other involved sites, and histopathological examination of tissue biopsies for visualization of yeast cells with the use of specific stains (12).

The existing literature on CLL patients diagnosed with disseminated histoplasmosis comprises 21 reports, with four of them noting that the fungal infection mimicked a recurrence of CLL (9, 11, 13–26). We present a rare case of a patient with chronic lymphocytic leukemia (CLL) who developed chronic disseminated histoplasmosis during the second decade of his illness. Subsequently, the patient experienced a RT transformation of his previously indolent CLL.

## Case presentation

A 65-year-old male was diagnosed with chronic lymphocytic leukemia/small lymphocytic lymphoma (CLL/SLL) in September 2002 following bone marrow examination with flow cytometry demonstrating a CD5-positive, CD23-positive monotypic B-cell population. At presentation, he had diffuse lymphadenopathy, splenomegaly, and left orbital involvement resulting in proptosis. He received localized radiation therapy to the left orbit prior to systemic treatment. Cytogenetic testing later identified isolated deletion of chromosome 13q, and immunoglobulin heavy chain variable region (IGHV) analysis revealed borderline-mutated status.

The patient achieved a complete response after six cycles of fludarabine and cyclophosphamide administered between October 2002 and March 2003, followed by rituximab consolidation. He experienced disease relapse in April 2008 with progressive lymphocytosis, adenopathy, anemia, thrombocytopenia, and B symptoms, prompting treatment with bendamustine and rituximab, resulting in a radiographic and hematologic complete response. A subsequent relapse occurred in March 2012 with symptomatic progressive disease, treated with six cycles of fludarabine, cyclophosphamide, and rituximab (FCR), complicated by prolonged post-treatment cytopenias.

By September 2014, recurrent absolute lymphocytosis was again noted. Repeat molecular testing in March 2015 confirmed persistence of 13q deletion and borderline-mutated IGHV status. Progressive symptomatic disease with marked splenomegaly and extensive bone marrow involvement prompted initiation of R-CHOP chemotherapy in October 2015. Treatment was complicated by tumor lysis syndrome during the first cycle and neutropenic fever requiring prolonged hospitalization after the second cycle, leading to early discontinuation of therapy.

**Figure 1:**
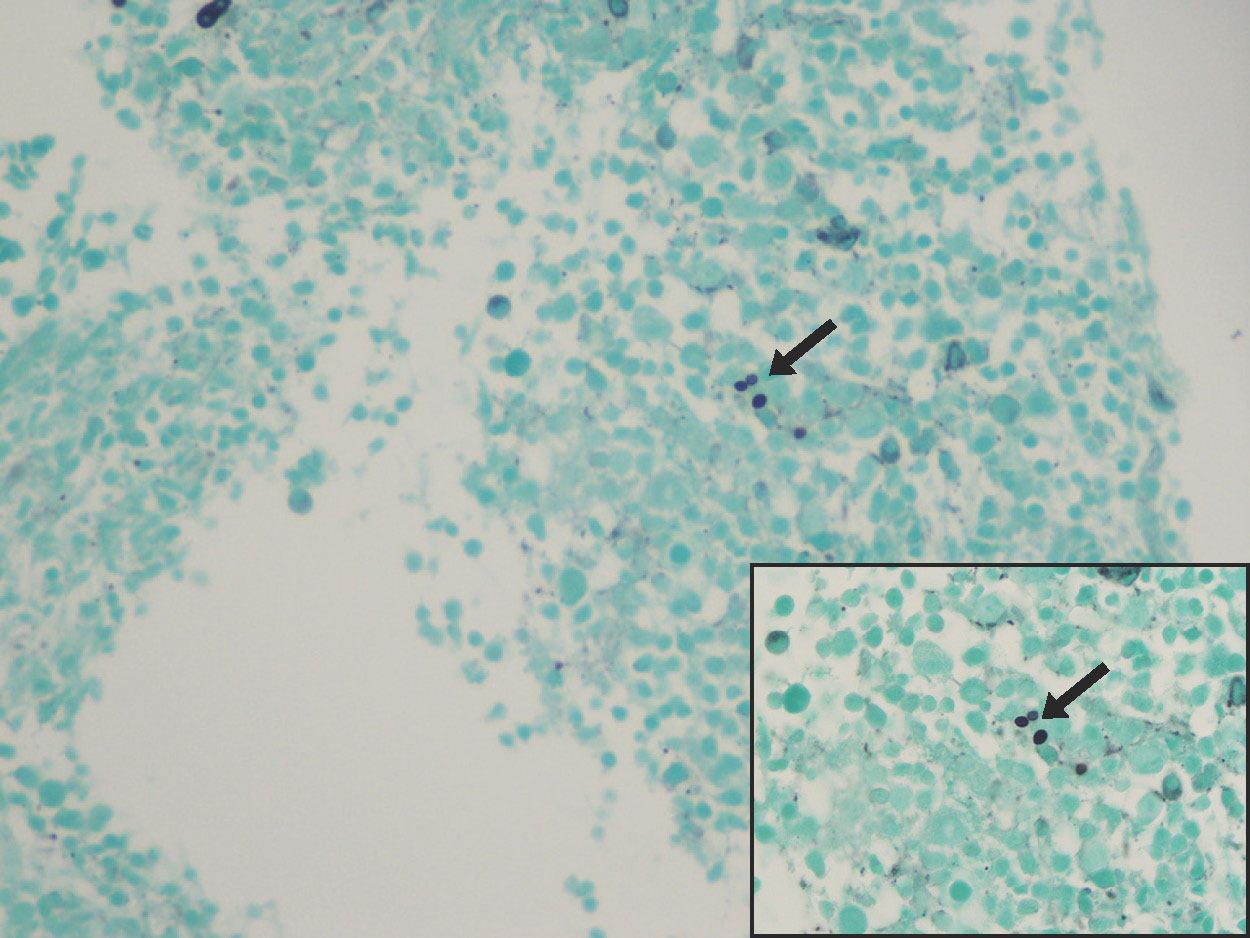
Bone marrow biopsy demonstrating Histoplasma capsulatum organisms, with magnified inset shown in the lower right panel.

In December 2015, bone marrow biopsy performed for evaluation of cytopenias revealed infiltration by Histoplasma capsulatum, consistent with disseminated histoplasmosis (**Figure 1**). The patient was treated initially with intravenous amphotericin B, followed by transition to itraconazole; however, due to intolerance, he required prolonged amphotericin therapy until May 2016. He subsequently began treatment with ibrutinib in March 2016, which he continued until February 2020.

In October 2019 (year 17 from initial diagnosis), the patient developed a newly enlarged left cervical lymph node and rising absolute lymphocyte count. PET/CT demonstrated a hypermetabolic left level 5B lymph node with a maximum standardized uptake value (SUVmax) of 17.5. Excisional lymph node biopsy in November 2019 revealed extensive necrotizing granulomatous lymphadenitis with rare fungal yeast forms morphologically consistent with Histoplasma species on Grocott methenamine silver staining, while acid-fast bacilli staining was negative. Concurrent flow cytometry demonstrated a monotypic lambda light chain–restricted B-cell population co-expressing CD5, CD20 (dim), and CD23, consistent with persistent involvement by the patient’s known CLL/SLL. No evidence of large-cell transformation was identified. Antifungal therapy with voriconazole was initiated, and the patient completed treatment in December 2020.

In January 2020, progressive lymphocytosis prompted repeat bone marrow biopsy, which demonstrated marked involvement by CLL with decreased trilineage hematopoiesis. Cytogenetic analysis revealed loss of the Y chromosome, and fluorescence *in situ* hybridization confirmed persistent deletion of 13q. TP53 mutation testing was negative. PET/CT imaging in February 2020 showed regression of the previously hypermetabolic cervical lymphadenopathy (SUVmax decreased from 17.5 to 3.6), interval enlargement of retroperitoneal and periceliac lymph nodes with low-to-moderate FDG uptake, and increasing nodal size without significant metabolic activity in multiple regions. Venetoclax therapy was initiated with dose escalation beginning in March 2020, with brief overlap of reduced-dose ibrutinib to prevent disease flare. Venetoclax was continued at a reduced dose of 100 mg daily due to drug interactions and gastrointestinal intolerance.

In September 2022, (year 20 post CLL diagnosis), the patient presented with constipation, polyuria, joint pains, and fatigue. Laboratory results were significant for impaired kidney function, with a Glomerular Filtration Rate (GFR) of 44 mL/min/1.73 m² (normal range >90 mL/min/1.73 m², last GFR around 50) and creatinine of 2.43 mg/dL (baseline 1.63 mg/dL), elevated lactate dehydrogenase (LDH) of 281 U/L, as well as presumed malignant hypercalcemia of 11.2 mg/dL (normal levels 8.5-10.2 mg/dL). The patient had a normal PTHrP level (0.7 pmol/L), elevated 1,25-dihydroxyvitamin D (142 pg/mL), and a normal 25-hydroxyvitamin D level (50 ng/mL). Serum protein electrophoresis revealed hypogammaglobulinemia without monoclonal protein. These findings raised concern for malignancy-associated hypercalcemia versus granulomatous disease.

The patient was hospitalized for recurrent episodes of severe hypercalcemia (up to 15.5 mg/dL) with associated acute kidney injury and neurocognitive symptoms. Bone marrow biopsy performed in October 2022 demonstrated normocellular marrow (40–50%) with preserved trilineage hematopoiesis and abundant non-necrotizing granulomas. No morphologic or immunophenotypic evidence of lymphoma was identified, and GMS staining was negative for fungal organisms. He was treated with intravenous fluids, calcitonin, bisphosphonates, and corticosteroids, and antifungal therapy was resumed.

**Figure 2:**
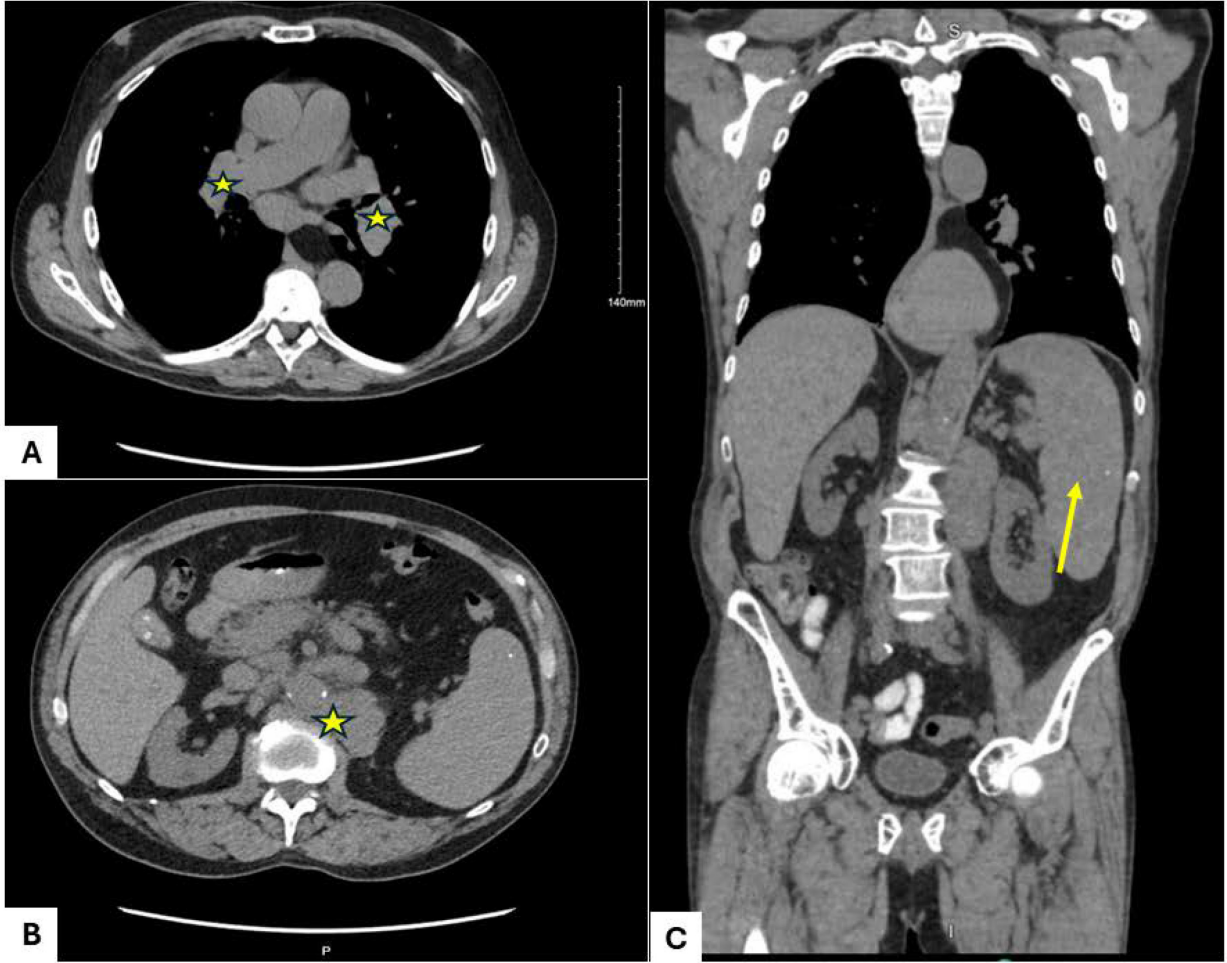
Computed tomography (CT) imaging: **(A)** Axial view demonstrating enlarged mediastinal lymph nodes (stars). **(B)** Axial view showing increased subcarinal adenopathy (star). **(C)** Coronal view demonstrating splenomegaly compared with prior imaging.

**Figure 3:**
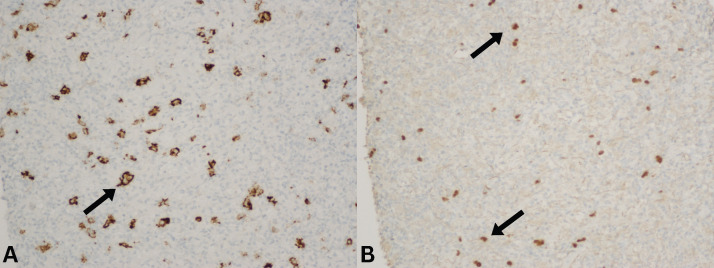
Retroperitoneal mass biopsy, histopathologic findings. **(A)** Biopsy section with atypical large cells positive for CD30 (arrow). **(B)** Biopsy section with atypical large cells showing dim PAX5 positivity (arrow).

In May 2023, the patient again developed marked hypercalcemia and acute kidney injury. CT imaging demonstrated significant progression of retroperitoneal lymphadenopathy, with a left periaortic lymph node measuring 8.9 × 7.8 cm, along with worsening splenomegaly (**Figure 2**). CT-guided core biopsy of the retroperitoneal lymph node performed in June 2023 revealed complete architectural effacement by a mixed lymphohistiocytic infiltrate containing scattered large atypical cells with lobulated nuclei and prominent nucleoli (**Figure 3**). Immunohistochemical staining demonstrated that the atypical large cells were strongly positive for CD30 and dimly positive for PAX5, while negative for CD15, CD20, CD45, and EBER *in situ* hybridization. The background lymphoid population consisted predominantly of T cells expressing CD3 and CD5 with mixed CD4 and CD8 expression and mildly diminished CD7. Ki-67 demonstrated increased proliferative activity in the atypical cells a. No clonal T-cell receptor gene rearrangement was detected, and no residual CLL/SLL was identified. These findings were diagnostic of classical Hodgkin lymphoma arising as Richter transformation from underlying CLL/SLL.

Patient was confirmed to have Stage IIIA cHL with subcarinal, paratracheal, periaortic lymphadenopathy. Adriamycin (doxorubicin), Vinblastine, and Dacarbazine (AVD) chemotherapy was initiated along with immunotherapy via nivolumab. At this point the patient was on a regimen of ibrutinib for approximately 4 years, it was discontinued due to clinical and radiographic evidence of disease progression. Given his worsening renal function, bleomycin was precluded. The patient has been monitored for a year and a half with stable-to-decreased size of his lymphomas and minimal chemotherapy-related neuropathy in the fingertips. Since then, the patient’s lymphomas are decreasing in size and his only symptoms are chemotherapy-related neuropathy of the fingertips. The patient remains on Venetoclax 100 mg daily and has no signs of active infection per their most infectious disease consultation. The patient completed a one-year course of antifungal therapy and was since discontinued due to lack of indication for lifelong treatment and the patient’s added side effects of chronic musculoskeletal pain. A CT scan from last month showed improved mediastinal adenopathy, stable adenopathy in the neck and abdomen, an unchanged spleen with no lesions, and no new suspicious findings in the chest, abdomen, or pelvis.

**Figure 4:**
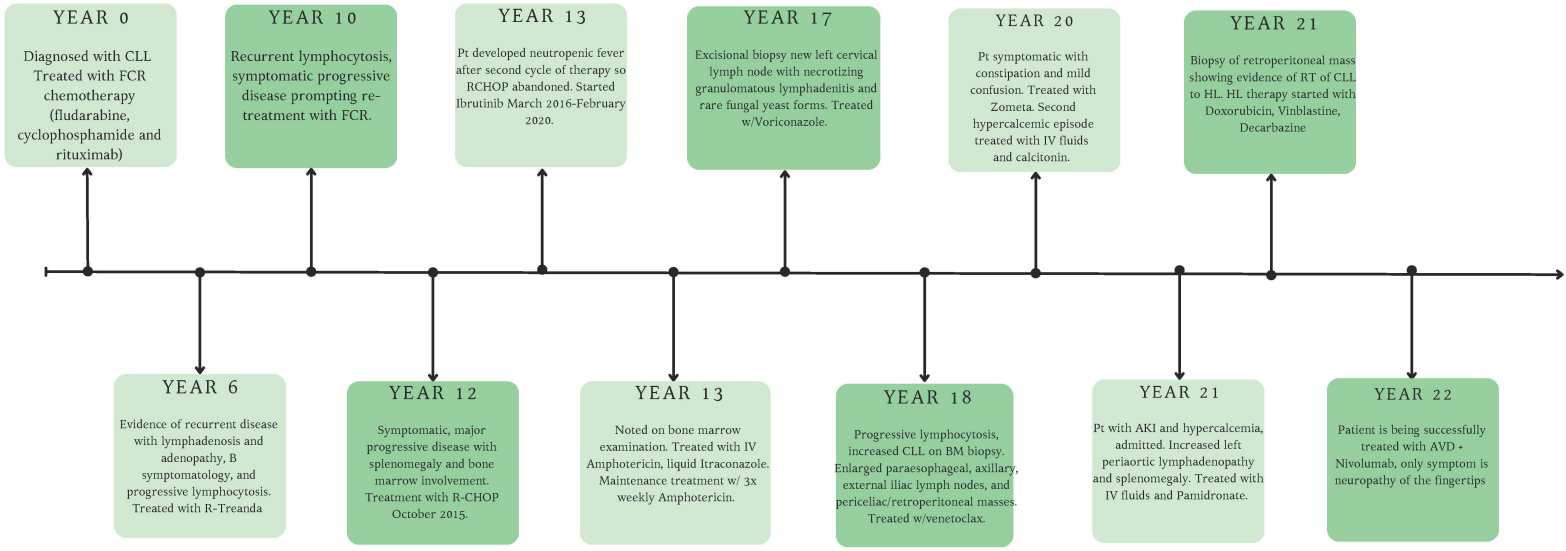
Timeline summarizing the patient’s clinical course, illustrating progression from chronic lymphocytic leukemia (CLL) to Hodgkin lymphoma (HL).

Given the patient’s extensive medical history and multiple hospitalizations, we have outlined the progression of their disease and corresponding treatments in a timeline format for clarity and completeness (**Figure 4**).

## Discussion

The rarity of transformation to Hodgkin’s lymphoma demands a high level of diagnostic precision and an informed treatment strategy (27). Further, characterizing the Richters Transformation (RT) as either an aggressive non-Hodgkin lymphoma or Hodgkin’s lymphoma is essential due to the distinct therapeutic approaches and prognostic outcomes associated with each (28). In evaluation of a suspected RT, patients may demonstrate rapid clinical decline, worsening B symptoms (fever, night sweats, weight loss), elevated LDH, and rapidly enlarging lymphadenopathy (29). According to several case reports, the presence of malignant hypercalcemia, typically defined as calcium above 14 mg/dL, in CLL patients should raise suspicion for RT (30–32). Four other case reports have discussed patients with hematologic malignancies transformed into a large B cell lymphoma after developing hypercalcemia from unknown etiology. Differentials in these cases also include humoral hypercalcemia of malignancy, PTHrp associated hypercalcemia, nonparathyroid neoplasia related to Hodgkin or non-Hodgkin lymphoma, or increased activity of osteoclasts (33). Other cases have also noted this type of hypercalcemia as a rare complication in patients with chronic disseminated histoplasmosis (CDH) (34, 35).

Acknowledging the fact that the patient had recurring CLL which required multiple treatments over the span of two decades, it is important to note that the treatment regimen patient went through with regards to chemotherapy would not be the same if he presented today. For example, before the widespread adoption of targeted therapies, chemoimmunotherapy regimens, including R-CHOP, were used for aggressive CLL variants, as in the case with this patient, or for Richter transformation to DLBCL, although FCR (fludarabine, cyclophosphamide, rituximab) and BR (bendamustine, rituximab) were more standard for CLL itself. R-CHOP was the standard of care for DLBCL-type Richter transformation, despite modest response rates and poor long-term outcomes in this setting (36).

The progression from CLL/SLL to Hodgkin’s lymphoma is fraught with uncertainties and compounded by underlying infections as consequences of immunosuppressive therapies (37). While the latter therapies may be avoided, the impact of CLL on the immune system is another non-modifiable complicating factor. Immunosuppression is inherent to the process of CLL with the most predominant defect being hypogammaglobulinemia, predisposing CLL patients to much higher rates of infection, and reinfections of otherwise indolent infections, compared to the general population. Studies have demonstrated derangements in levels of IgG, IgA, and IgM in CLL patients, a phenomenon also reflected in our patient as well (38). In addition, this population often face a prolonged risk of infections due to compromised B-cell and T-cell functions (16).

Prognosis for Hodgkin lymphoma as Richter transformation is intermediate: worse than *de novo* HL but better than DLBCL-type RT (39). Two-year overall survival is approximately 57–74% in population-based cohorts, with median survival ranging from 1.7 to 3.3 years (40). Adverse prognostic factors include anemia, elevated LDH, high International Prognostic Score, and poor performance status (40). Prior fludarabine exposure may worsen outcomes (2). The differential diagnosis includes progressive CLL, DLBCL-type RT, and Hodgkin-like lesions (isolated HRS cells in a CLL background). Histopathologic confirmation is mandatory, with classic HL morphology and immunophenotype (CD30+, CD15+, PAX5 weak, CD20–/dim). PET/CT can guide biopsy of the most metabolically active node. EBV is detected in a majority of cases, but its role is not fully defined. Clonality studies show that about 30–50% of cases are clonally related to the underlying CLL (4). Treatment options are based on standard HL regimens. The preferred first-line therapy is ABVD (adriamycin, bleomycin, vinblastine, dacarbazine), with curative intent in fit patients. Two-year progression-free and overall survival rates with ABVD/ABVD-like regimens are 70% and 74%, respectively, in real-world cohorts (27, 36, 39).

While an elevated risk of viral infections and reactivations is well-documented, atypical presentations of histoplasmosis in the context of CLL has rarely been reported and can be clinically indistinguishable from a RT (9). Our case featuring a patient with generalized signs and symptoms preceding such transformation demonstrates the challenge of differentiating the two diagnoses due to several overlapping findings such as lymphadenitis and B-symptomatology. Our patient presented with necrotizing granulomatous lymphadenitis with rare fungal yeast forms and a left cervical adenopathy status following a remote yet recurrent disseminated infection with histoplasmosis in the bone marrow. Shortly after, our patient was discovered to have a CLL transformation to Hodgkin’s lymphoma.

Histoplasmosis treatment depends on disease severity and immune status. Mild cases in immunocompetent patients may not require therapy, but moderate or worsening disease is typically treated with itraconazole (41). Severe or disseminated cases, especially in immunocompromised individuals, require induction with liposomal amphotericin B followed by long-term itraconazole (41). Alternatives like voriconazole or fluconazole may be used if itraconazole is not tolerated.

The divergence from the expected presentations of disseminated and pulmonary histoplasmosis to lymphadenopathies in 4 out of 21 reported histoplasmosis cases in CLL patients, as shown in **Table 1**, raises the question of whether such infections could be linked to CLL transformations (9, 11, 13–26). In fact, one case demonstrated a lymphadenopathy in the mediastinum, in a patient who also developed transformation to a T-cell lymphoma (20). The role of other infections such as Epstein-Barr Virus (EBV) have been further studied and linked to transformation to a more aggressive lymphoma, particularly following treatments like Fludarabine that diminish T-lymphocyte populations, and perhaps inadvertently foster an environment ripe for the proliferation of EBV-infected B-cells (2, 42). Though no definitive link outside of EBV infection has been established, the parallels drawn with previously reported cases raise the question on whether there could be a link between fungal infections and CLL transformations. Our case, of a CLL patient, with low immunoglobulins and eventual RT following several lymph nodal reinfections by histoplasmosis, further intensifies the inquiry of whether CLL infection and/or treatment in CLL patients may play a role in an eventual RT. In patients with recurrent infections and hypogammaglobulinemia, immunoglobulin replacement therapy has been shown to reduce infection frequency and improve quality of life. Moreover, our case addresses the question of hypercalcemia and its potential role in the process of RT from CLL to cHL. Our patient’s hypercalcemia may have been related to his development of histoplasmosis, or could be related to the RT, these two concomitant diagnoses certainly complicate the diagnosis process. Evidently, this highlights the need for deeper exploration into the possible link between disseminated histoplasmosis and RT and their shared effect of hypercalcemia, as well as how this relates to the progression of CLL to cHL. From a clinical standpoint, emphasis should be placed on patient education for potential signs of transformation, proactive reporting of symptoms, and potentially prophylactically treating for certain opportunistic infections. Lastly, provider-patient conversations regarding itraconazole prophylaxis may be considered to reduce rate of histoplasmosis and cryptococcus infections, although have not shown mortality benefit (43).

**Table 1:** Previously reported cases of chronic lymphocytic leukemia (CLL) associated with histoplasmosis.

First author (Year)	Study type	CLL treatment history	IgG level reported	Site of histoplasmosis	Diagnostic modality	Mimicked CLL progression	Additional notes
Jain et al. (9)	Case report	FCR and alemtuzumab	Not reported	Bone marrow, disseminated	Bone marrow biopsy, fungal stains	Yes	Pseudo–Richter transformation
Milkowski et al. (19)	Case report	Chlorambucil & Prednisone: 9 Cycles	Not reported	Disseminated	Bronchoscopy, biopsy	Yes	Richter Transformation to T-cell subtype large cell lymphoma amid disseminated histoplasmosis
Oliveira et al. (20)	Case report	FC (6 cycles) followed by FCR (6 cycles)	Not reported	Mediastinal nodes, lung	Biopsy	Yes	T-cell lymphoma transformation
Shahani (22)	Case report	FCR	Not reported	Disseminated	Imaging + biopsy	Yes	Mimicked relapse
Anonymous (14)	Case report	Phosphorus-32 (P-32)	Not reported	Disseminated	Autopsy, histopathology	Yes	Early-era report, limited data
Kauffman et al. (11)	Case series	Variable: cyclophosphamide, vincristine, prednisone, +/- procarbazine & chlorambucil	Not reported	Disseminated	Culture, Biopsy	Yes	Mixed malignancies included
Fulkerson et al. (16)	Case report	Cyclophosphamide and Prednisone	Not reported	Disseminated	Tissue biopsy	Yes	Opportunistic infection context
Johnston et al. (18)	Case report	Untreated	Not reported	Skin, lymph nodes	Biopsy with fungal stains	Yes	Cutaneous involvement
Torres et al. (24)	Case series	Mixed cancer therapies	Not reported	Lymphadenitis	Culture, Biopsy	Variable	Cancer hospital cohort
Xu et al. (26)	Case report	Chlorambucil (1 cycle)	Not reported	Disseminated	Peripheral blood film	Yes	Rare diagnostic modality
Frei et al. (15)	Cohort	Ibrutinib-treated	Not applicable	Invasive fungal infections	Registry-based	Not specified	Focus on IFIs, not individual cases
Schoen et al. (21)	Cohort	Pre- and post-treatment	Not applicable	Fungal infections	Database review	Not specified	Lacked organism-level detail
Fung et al. (17)	Cohort	Bendamustine	Not applicable	Disseminated	Claims-based analysis	Not specified	Infectious complications including histoplasmosis but not organism specific data
Silva et al. (23)	Case report	Untreated SLL	Not reported	Disseminated	Biopsy, culture	Yes	Infection preceded malignancy
Tsitsikas et al. (25)	Case report	Not reported	Not reported	Pulmonary	Imaging + biopsy	Yes	Diagnostic ambiguity emphasized

FCR = Fludarabine, Cyclophosphamide, & Rituximab.

## Conclusion

The overlapping presentation of RT and atypical infections point to the necessity of vigilant monitoring and management of opportunistic infections like histoplasmosis in cancer patients. This is especially significant, considering their frequent coincidence with later malignant transformation processes, highlighting a potential correlation between opportunistic infections and oncogenic progression. Beyond routine labs and imaging, CLL patients should be well-educated on their risks of acquiring opportunistic infections and thus advised to avoid risky behavior and locations. In this context, CLL patients should be encouraged to avoid contact with birds, their feces, caves, and abandoned pool houses and garages. Further translational studies are warranted to further analyze the effect of chronic histoplasmosis infections in lymph nodes and possible role in propagating RT.

## Patient perspective

The patient expressed satisfaction with the care he has received. He noted that he has been driving over two hours from his residence to the Bronson Cancer Center for each visit and emphasized that the experience has always been worth his time. In comparison to his symptoms of fatigue and abdominal pain upon initial presentation, he now only complains from musculoskeletal pain as a side effect of his maintenance medications. He also shared his hope that his medical history and diagnoses can be used to help train future medical professionals.

## Data Availability

The original contributions presented in the study are included in the article/supplementary material. Further inquiries can be directed to the corresponding author.
